# Human Pancreatic Islet Isolation: Part II: Purification and Culture of Human Islets

**DOI:** 10.3791/1343

**Published:** 2009-05-26

**Authors:** Meirigeng Qi, Barbara Barbaro, Shusen Wang, Yong Wang, Mike Hansen, Jose Oberholzer

**Affiliations:** Department of Surgery, University of Illinois, Chicago

## Abstract

Management of Type 1 diabetes is burdensome, both to the individual and society, costing over 100 billion dollars annually. Despite the widespread use of glucose monitoring and new insulin formulations, many individuals still develop devastating secondary complications. Pancreatic islet transplantation can restore near normal glucose control in diabetic patients ^1^, without the risk of serious hypoglycemic episodes that are associated with intensive insulin therapy. Providing sufficient islet mass is important for successful islet transplantation. However, donor characteristics, organ procurement and preservation affect the isolation outcome ^2^. At University of Illinois at Chicago (UIC) we developed a successful isolation protocol with an improved purification gradient ^3^. The program started in January 2004 and more than 300 isolations were performed up to November 2008. The pancreata were sent in cold preservation solutions (UW, University of Wisconsin or HTK, Histidine-Tryptophan Ketoglutarate) ^4-7^ to the Cell Isolation Laboratory at UIC for islet isolation. Pancreatic islets were isolated using the UIC method, which is a modified version of the method originally described by Ricordi *et al*^8^. As described in Part I: Digestion and Collection of Pancreatic Tissue, human pancreas was trimmed, cannulated, perfused, and digested. After collection and at least 30 minutes of incubation in UW solution, the tissue was loaded in the cell separator (COBE 2991, Cobe, Lakewood, CO) for purification ^3^. Following purification, islet yield (expressed as islet equivalents, IEQ), tissue volume, and purity was determined according to standard methods ^9^. Isolated islets were cultured in CMRL-1066 media (Mediatech, Herndon, VA), supplemented with 1.5% human albumin, 0.1% insulin-transferrin-selenium (ITS), 1 ml of Ciprofloxacin, 5 ml o f 1M HEPES, and 14.5 ml of 7.5% Sodium Bicarbonate in T175 flasks at 37°C overnight culture before islets were transplanted or used for research.

**Figure:**
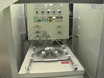


## Protocol

### 1. Purification of islets

To start the islet purification procedure, load the COBE bag, clamp all tubes except the green tube, and set the speed to 1,500 and the super out to 0.
In the hood set up the pump and gradient beakers and connect the collection tubing to the green tube.
Now the loading of the ficoll 1.100 can begin: push “spin” then add 110 ml of Ficoll 1.100 to the front beaker, and start the pump in order to load the COBE bag.  As the Ficoll is loaded, press super out, stop the pump, unclamp the pump head and set the super out speed to 100.
When the ficoll reaches the beaker and all air is expelled from the tubing, re-clamp at pump head. Set the spin speed to 3,000, and the super out speed to 0.
Now prepare to load the gradients by pouring the heavy gradient into the front beaker.  Slightly release the clamp between the two beakers to remove any air.  Then pour the light gradient into the back beaker.
Turn on the magnetic stirrer, press “spin” on the COBE machine, remove the clamp from the tube connecting the two beakers, and visually verify that the heavy and light gradients are mixing.
Once the gradients started mixing, turn on the centrifuge. Wait one minute, and then turn on the pump to load the mixing gradients.
To load the tissue let the gradient run low in the front beaker, but not low enough to allow air to enter the loading tube, then reclamp the tube connecting the beakers and load the tissue.
Continue adding the rest of the tissue.  Then wash the tube that held the tissue with 50 ml of wash solution, and use it to wash the front beaker.
As the last of the tissue enters the bag, simultaneously stop the pump, unclamp at the pump head, and clamp tubing above the bag. Continue to centrifuge for 5 minutes.
While centrifugation continues, bring the 12 collection tubes to the hood and loosen their caps. Attach the collection tubing to the yellow tube, and place the sterile collection tip into conical tube 1.  Then unclamp the yellow tube, and clamp the green tube.
When the spin ends, slowly raise Superout to 100. Collect 150 ml in tube 1. Then collect 30 ml in tubes 2-12 (from 200-230 ml).
Once collection is complete, push “STOP” on the COBE.  Now that purification has ended, the islets can be assessed and cultured.

### 2. Sampling and culture

After collecting the purified islets fractions, remove a sample from each of the 12 collection tubes and stain with Dithizone.  The staining will show which are layers contain high purity islets vs. layers with low purity islets.
Collect the high and low purity tissue and pool each together.
Spin and wash twice. After the second wash, bring the volume of each to 250 ml with Final Wash media.
To assess the pooled fractions start by taking two 1 ml samples from the high of the pooled fractions and one 1 ml sample from the low.  Transfer each 1ml sample into a conical tube containing 9 ml of wash solution.
Then transfer 1 ml from each 15 ml tube to a counting dish. Examine and count the cells under the microscope.
After counting the cells calculate the number of flasks needed to culture the islets using the following formula: (total EIN / purity of islets in decimal) / (30,000 EIN per flask). For example, 100,000 islets with 90% purity are cultured in 4 flasks.
Transfer the islets to T175 flasks and culture in a 37˚C and 5% CO_2_ incubator.

## Discussion

In spite of significant advancements in techniques of human islet isolation, islet yield remain highly variable and unpredictable. Purification of digested pancreatic tissue is crucial to recover a sufficient isle mass after successful enzymatic digestion for transplantation. The UIC purification method is recommended because a superior recovery of highly pure human islets was demonstrated by using this method. Moreover, up to 50 ml of digested tissue can be loaded in a single Cobe run for purification, thus minimizing the ischemia-associated injury of the human islets by shortening the total time used on isolation.
